# A Competency Framework for Medical AI Education: Mixed Methods Study

**DOI:** 10.2196/91116

**Published:** 2026-05-20

**Authors:** Chang Cai, Gaoxia Zhu, Shang-Ming Zhou, Olivia Ng, Jamie Duell, Weng Kin Ho, Daisy Minghui Chen, Bernett Lee, Fang Li, Siyuan Liu, Vidya Sudarshan, Li Rong Wang, Chanwoo Choi, Xiuiyi Fan

**Affiliations:** 1 Lee Kong Chian School of Medicine, Nanyang Technological University, 50 Nanyang Avenue, Singapore, Singapore, 65 82634539; 2 National Health Group, Singapore, Singapore; 3 National Institute of Education, Nanyang Technological University, Singapore, Singapore; 4 Center for Health Technology, Faculty of Health, University of Plymouth, Plymouth, United Kingdom; 5 School of Computing and Digital Technologies, Sheffield Hallam University, Sheffield, United Kingdom; 6 Centre of Excellence in AI and Robotics, Sheffield Hallam University, Sheffield, United Kingdom; 7 Singapore Immunology Network (SIgN), Agency for Science, Technology and Research (A*STAR), Singapore, Singapore; 8 Infectious Disease Labs (ID Labs), Agency for Science, Technology and Research (A*STAR), Singapore, Singapore; 9 College of Computing and Data Science, Nanyang Technological University, Singapore, Singapore; 10 Centre for Frontier AI Research, Agency for Science, Technology and Research (A*STAR), Singapore, Singapore; 11 Centre of AI in Medicine, Nanyang Technological University, Singapore, Singapore

**Keywords:** artificial intelligence, AI, AI competency framework, AI literacy, AI training, medical education, medical professionals, United Nations Educational, Scientific and Cultural Organization, UNESCO

## Abstract

**Background:**

Although artificial intelligence (AI) is increasingly adopted in health care, clinicians face barriers, including insufficient understanding, limited trust, and challenges in interpreting AI outputs. Existing frameworks, such as the United Nations Educational, Scientific and Cultural Organization (UNESCO) AI competency framework, lack clinical specificity. Additionally, there remains limited evidence on framework-based training programs for medical professionals.

**Objective:**

This study aimed to (1) develop a medical AI competency framework and (2) apply the framework to design an AI training program and pilot 1 module.

**Methods:**

We conducted a mixed methods study comprising 2 phases. In Study 1, we developed a medical AI competency framework by integrating the UNESCO AI framework with the Miller pyramid model. The framework was refined through expert input from 24 stakeholders (6 hospital administrators, 8 medical professionals, and 10 university instructors). Expert responses were analyzed using deductive content analysis based on predefined codebooks. In study 2, we designed an AI training program based on the framework, and evaluated it using a 2-round Delphi process. Nine educators with expertise in instructional design, medical education, and AI participated in the Delphi study. Consensus in round 1 was defined as an IQR≤1, agreement score proportion >75%, and full-score frequency >49.23%; in round 2, consensus was defined as agreement score proportion >80%. A pilot workshop with 28 participants and 4 instructors assessed the feasibility of 1 module using self-reported measures of satisfaction, engagement, and confidence.

**Results:**

A 6D 4-level medical AI competency framework was developed. Among the 24 experts, 19 (79.17%) mentioned competencies related to AI foundations, and 23 (95.83%) mentioned competencies related to application skills. The framework was translated into a 5-module training program, covering patient-centered and ethical AI, privacy and security, medical AI applications, bias and health equity, and generative AI in health care. Each module included 5 elements: content, learning goals, teaching activities, learning resources, and assessment. The Delphi process achieved complete consensus across all 25 elements of the training program. The pilot workshop indicated high participant satisfaction (mean 4.00, SD 0.52), good engagement (mean 3.80, SD 0.71‐mean 4.05, SD 0.51), and moderate self-reported confidence (mean 3.63, SD 0.53). These findings suggest that the module was feasible, although outcomes should be interpreted cautiously given the self-reported and short-term nature of the evaluation.

**Conclusions:**

The framework provides a structured reference for AI training program design in medical education. The workshop findings provide preliminary support for the feasibility of the program’s technical module, while also highlighting the need for broader and longer-term evaluations. Future work should expand the framework and training program to new regions and delivery formats (eg, semester-long courses and continuing medical education) and evaluate their long-term impact.

## Introduction

Artificial intelligence (AI) is transforming health care by enabling early diagnosis of diseases, real-time health monitoring, and personalized health care [1-4]. It also contributes to health care management by enhancing patient satisfaction and optimizing hospital resources [5]. Nevertheless, clinicians encounter multiple barriers to AI adoption, including risks of misuse, insufficient understanding, limited trust in AI, and challenges in interpreting AI outputs [6,7].

These challenges underscore the need for clinicians to acquire AI competencies, as *“*AI is not meant to replace medical professionals, but the ones using AI will probably replace those who don’t*”* [8, page 4]. Additionally, responsible AI adoption depends not only on external safeguards [9] but also on the capacities of medical professionals to interpret, evaluate, and oversee AI use in health care [10-16].

However, a dedicated AI competency framework tailored to medical professionals remains lacking. Global initiatives, such as the United Nations Educational, Scientific and Cultural Organization (UNESCO)’s AI competency framework [17], offer general guidelines for AI literacy but do not address the specific requirements of clinical practice. AI in health care requires unique considerations, including patient-centered decision-making, data governance, and ethical responsibility [11,12,18-20]. For example, clinicians need to explain AI-supported recommendations to patients, assess the reliability and limitations of AI outputs, and ensure that the use of AI aligns with patient values [10-12]. Moreover, proposed health care–related AI competencies are often limited to specific specialties, educational levels, or traditional AI technologies [13,15]. Emerging technologies, such as generative AI (GenAI), further highlight the insufficiency of existing proposed AI competencies. Although GenAI has considerable potential for health care [21], it also introduces challenges, including hallucinations and source uncertainty [22,23]. These challenges differ from those of traditional AI systems and require new competencies, such as verifying AI-generated content and ensuring clinician oversight [21,24]. However, few studies have examined the specific competencies required for medical professionals to use GenAI safely and effectively.

Furthermore, the absence of a dedicated competency framework has implications for medical education. Existing medical AI education initiatives, such as extracurricular workshops, online courses, and specialized lectures [25-29], often focus on specific topics rather than a shared foundational competency structure. As a result, there is limited guidance on how AI skills should be developed, assessed, and integrated into curricula. Recent reviews have highlighted this gap, noting the lack of framework-guided curriculum design in medical AI education [30,31]. More broadly, AI education should not be limited to technical content coverage alone. It also requires structured pathways and guidance to help learners address application risks and support responsible implementation [32].

These gaps highlight the need for a comprehensive, clinically grounded, and future-oriented medical AI competency framework. Such a framework should be directly applicable to curriculum development. To address these issues, this study aims to answer the following research questions:

- What core AI competencies should medical professionals possess to meet the evolving demands of AI-enabled health care?
- How can we use an AI competency framework to build a training program?

This study makes 3 contributions. First, we develop a medical AI competency framework, aligning AI competencies with clinical demands. Second, we showed the practical application of the framework by designing an AI training program. Third, we provide preliminary evidence of feasibility through pilot implementation, offering insights into the design and delivery of AI curricula in medicine. This research may contribute to the integration of AI into medical education and practice.

A preliminary version of this work was presented in the Artificial Intelligence in Education 2025 conference proceedings [33]. That paper reported on the development and refinement of the medical AI competency framework. This study substantially extends that foundational work in several important ways. First, it provides a more detailed and rigorous description of the framework development and expert-informed refinement process. Second, it extends the framework by incorporating observable behaviors, making it more actionable for teaching and assessment. Third, building on the framework, a training program for medical AI education was developed and evaluated through a 2-round Delphi process. Finally, a pilot study of 1 module of the program was implemented, and student feedback was collected to examine its feasibility. Overall, this study extends the preliminary work by providing a more actionable framework, showing its application in curriculum development, and offering preliminary evidence of implementation feasibility through a pilot module.

## Methods

### Overview

**Figure 1.**
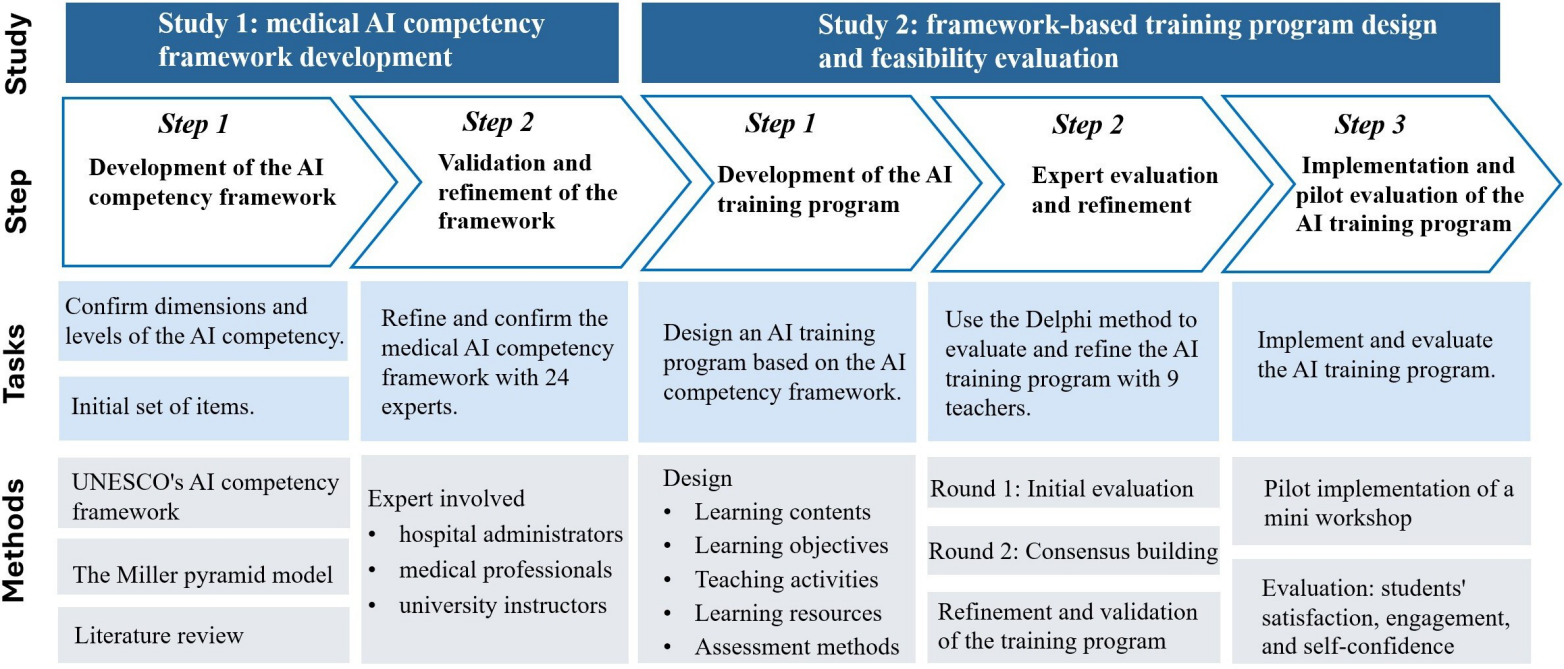
Five-step development of the medical artificial intelligence (AI) competency framework and training program. UNESCO: United Nations Educational, Scientific and Cultural Organization.

This research consists of 2 studies, as shown in Figure 1. Study 1 focused on the development of the medical AI competency framework. An initial version of study 1 was reported in our Artificial Intelligence in Education 2025 conference paper [33]; in this paper, this component is described in greater methodological detail, while study 2 is newly reported. Study 2 showed one practical application of this framework. We first designed a training program grounded in the competency framework. The program was subsequently evaluated and refined through a 2-round Delphi process. Finally, a mini workshop was carried out to examine the program’s feasibility.

### Study 1: Development of the Medical AI Competency Framework

#### Step 1: Initial Medical AI Competency Framework Conceptualization

This step establishes the theoretical foundation for the initial medical AI competency framework. The framework was developed based on (1) UNESCO’s AI competency framework [17], (2) the Miller pyramid [34], and (3) prior literature on AI competencies in medicine [11,12,15,35,36].

##### Theoretical Foundation

To conceptualize medical AI competence, we first drew on UNESCO’s AI competency framework [17], a general guideline for AI literacy development. It outlines four core domains [17]: (1) human-centered mindset (developing a human-focused perspective on AI’s benefits, risks, and proportionality in addressing societal and environmental needs), (2) ethics of AI (acquiring social and ethical competencies to guide the responsible use of AI throughout its lifecycle), (3) AI techniques and applications (combining conceptual understanding with practical skills through the real-world application of AI tools), and (4) AI system design (developing technical proficiency in designing, implementing, and refining AI systems).

Building on these domains and prior literature [11,12,15,17,35,36], we derived 6 competency dimensions tailored to the needs of health care practice. From UNESCO’s *human-centered mindset*, we introduced *patient-centered AI in health care* to capture clinicians’ ability to interpret and apply AI-supported decisions while preserving autonomy and maintaining human oversight. Extending UNESCO’s *ethics of AI*, we proposed *ethics and transparency in clinical AI*, highlighting ethical principles in medical AI implementation. From UNESCO’s *AI techniques and applications*, we derived *technical proficiency in medical AI applications*, emphasizing practical AI use by clinicians. Integrating UNESCO’s *AI system design* with a focus on health care equity [37,38], we introduced *bias mitigation and health equity in AI design*. Considering the importance of data privacy and governance in medicine [19,20], we incorporated *data privacy, security, and compliance in health care AI*. Finally, recognizing the expanding role and challenges of GenAI in health care [21-23], we proposed *GenAI for health care*.

##### Proficiency Levels

Rather than UNESCO’s 3-level progression (understand, apply, and create), we adopted the Miller pyramid model [34] to guide competency development. Unlike generic progression models, the Miller pyramid provides a clinically oriented structure that differentiates knowledge, application, simulated performance, and real clinical practice [34], making it well-suited for medical professionals. Widely used in medical education to assess clinical skills [39,40], it defines stepwise progression for competency development. The Miller pyramid includes the following levels [34]:

1. Know (knowledge): acquisition of foundational knowledge.
2. Know how (competency): the ability to apply knowledge in context.
3. Show how (performance): demonstration of skills in simulated settings.
4. Do (action): application of skills in real clinical practice.

These 6 dimensions and 4 levels jointly constituted the initial medical AI competency framework (Table S1 in Multimedia Appendix 1).

### Step 2: Expert-Informed Framework Refinement

In this step, the initial framework was used to build deductive codebooks (Tables S2-S7 in Multimedia Appendix 1). The codebook was used to analyze experts’ responses to the open-ended questions.

#### Expert Panel

To examine whether the initial framework aligned with expert views and to refine it where needed, a total of 24 experts from Singapore were selected, including 6 hospital administrators, 8 medical professionals, and 10 university instructors (Table 1). These diverse stakeholders ensured alignment of the framework with health care system needs and pedagogical principles. The selection of Singaporean experts was due to logistical constraints, and future studies will expand to include international perspectives.

**Table 1.** Demographics and selection criteria of framework validation experts.

Experts	Number of experts	Department	Selection criteria
Hospital administrators	6	Hospitals and government agencies	Leaders from hospitals or government agencies with more than 5 years of experience in policymaking and management.
University instructors	10	A university and health care organization	Teachers from universities or health care organizations with more than 5 years of teaching experience in AI^a^ or medicine.
Medical professionals	8	Hospitals	Medical professionals with more than 5 years of clinical experience and prior exposure to AI applications.

a AI: artificial intelligence.

#### Data Collection

Experts were invited via email between August 2024 and December 2024 and completed an anonymous online survey with open-ended questions. Responses were collected anonymously to ensure participant confidentiality. Participants had the opportunity to review and modify their responses before submission.

The survey included the following questions for all experts:

- What AI-related skills do you think medical professionals should acquire?
- What topics in AI for medicine, do you consider essential for medical professionals to learn?
- For each AI-related skill mentioned above, to what extent do you think medical professionals should possess it (eg, basic awareness, practical knowledge, high-level skills, or advanced expertise)?

For medical professionals, additional questions included the following:

- Based on your own work experience, how can AI complement medical practices?
- What are the main barriers you have encountered when using or attempting to use AI?
- What challenges have you faced, or do you anticipate facing, when co-designing and co-developing a medical AI product?

#### Data Analysis

Expert feedback was analyzed through deductive content analysis using predefined codebooks (Tables S2-S7 in Multimedia Appendix 1). The codebooks were developed based on the initial medical AI competency framework (Table S1 in Multimedia Appendix 1), which was derived from our prior work [33]. Two researchers independently coded one-third of the responses using the codebooks to assess inter-rater reliability. The κ coefficient was 0.81, indicating strong agreement [41]. Researchers assigned each response to the most relevant competency category. Comments that did not fit existing definitions or suggested a new competency were flagged, discussed iteratively, and refined. In this study, we did not identify expert comments that justified adding a new dimension. However, several code definitions were refined to better reflect the expert comments while preserving the broader 6D structure. Discrepancies were resolved through iterative discussion until full consensus was reached. After establishing reliability, 1 researcher coded the remaining responses using the codebooks.

### Study 2: Framework-Based Training Program Design and Feasibility Evaluation

#### Step 1: Framework-Based AI Training Program Development

We developed an AI training program as an application of the framework. Prior AI in Medicine initiatives suggested that combining lectures with case-based learning, hands-on activities, and problem-based learning supports the application of AI concepts in clinical contexts and enhances learner engagement [25,26,42,43]. Additionally, flipped classrooms have been extensively adopted to improve learning outcomes [25,44]. Integration of online and offline resources can support teaching design by aligning preclass learning, in-class interaction, and practice [45]. To support experiential and reflective learning, the Kolb experiential learning cycle and the Gibbs reflective cycle were introduced [46,47]. These pedagogical and theoretical principles informed the design of the program’s learning activities and assessment strategies.

The program design included 5 core components: content, learning objectives, teaching activities, learning resources, and assessment methods. Modules were aligned with the competency framework, and learning objectives were mapped to the Miller pyramid. We also adopted teaching activities aligned with the Miller pyramid. Lectures build foundational knowledge (know); case studies, discussions, and hands-on activities enable application and demonstration (know how and show how); and problem-based learning fosters real-world problem-solving (do). Various resources were provided: (1) curated datasets for hands-on data analysis, (2) case studies on AI’s clinical application, and (3) online platforms for coding.

#### Step 2: Evaluation and Refinement of the Training Program

To validate the AI training program, we used the Delphi method, inviting 9 educators with expertise in AI, medical education, and instructional design.

##### Course Evaluation Panel

The panel was selected based on three criteria: (1) participants have designed at least 1 course, (2) participants have teaching experience in higher education, and (3) participants are from the fields of education, medicine, or AI.

As detailed in Table 2, the course evaluation panel included 2 education specialists from the education department of a public university in Singapore, 3 AI teachers from the computer science department of a public university in Singapore, and 4 medical teachers from the medical departments of public universities in Singapore and the United Kingdom. This multidisciplinary composition ensured a balanced integration of pedagogical, AI, and clinical expertise, providing a comprehensive evaluation of the training program.

**Table 2.** Demographics of the course evaluation panel participant.

Participant	Number of experts	Department	Nationality
Education experts	2	Educational department	Singapore
AI^a^ educators	3	Computer science department	Singapore
Medicine educators	4	Medicine department	Singapore and the United Kingdom

a AI: artificial intelligence.

##### Delphi Method

The Delphi method is a well-established, anonymous group process designed to achieve consensus among participants [48,49]. This method has been widely used in previous studies to support and validate course design [50-52]. We conducted a 2-round Delphi process over 1 month to reach consensus on the AI training program design. No consultation on methods took place. Participants were invited by email and completed online questionnaires. The process followed a quasi-anonymous design: panelists were not informed of each other’s identities, and no direct interaction occurred between participants. Responses were deidentified prior to analysis.

In round 1, participants reviewed the proposed 5-module training program and rated 25 items (covering content, learning objectives, teaching activities, learning resources, and assessment) using a 5-point Likert scale (1=“strongly disagree” to 5=“strongly agree”). Open-ended comments were also collected to inform revisions. Qualitative feedback from round 1 was synthesized by 1 researcher by deduplicating and grouping similar suggestions. The grouped suggestions were then summarized into specific revisions (eg, module, element, and corresponding suggestions). A second researcher reviewed the synthesized feedback, and discrepancies were resolved through discussion. The aggregated feedback was then provided to the same panel in round 2. Participants re-evaluated the revised items based on the summarized feedback from round 1 using the same rating scale. The round 1 and round 2 surveys can be accessed in Multimedia Appendix 2.

##### Data Analysis and Consensus

Panel ratings were analyzed using the IQR, agreement score (AS) proportion (proportion of ratings ≥4), and full-score (FS) frequency (proportion of ratings=5), consistent with previous studies [52-57]. All consensus thresholds were prespecified. In round 1, an item was considered to have reached consensus if it met all 3 criteria: IQR ≤1, AS >75%, and FS above a predefined threshold. Following previous work [58], the threshold for FS was calculated as mean–SD, which yielded 49.23% (63.11%-13.88%). In this study, FS>49.23% corresponds to more than half of the panel assigning the highest score. Items that did not meet these criteria were carried forward to round 2. In round 2, consensus was defined as achieving AS>80% [55-57].

### Step 3: Pilot Implementation of a Mini Workshop

To assess the feasibility of the designed AI training program, we conducted a pilot mini workshop. Due to time constraints, the workshop focused primarily on module 3 (medical AI applications), while the remaining modules remained at the design stage and were not fully implemented. Module 3 was selected because it represented the core technical component of the program. A 1-day in-person workshop was held in July 2025. A total of 28 participants and 4 instructors attended. During in-class sessions, participants worked in small groups to analyze real-world AI use cases in health care. The teaching activities included lectures, hands-on sessions, group presentations, and reflective discussions.

After the workshop, participants were invited to complete a voluntary and anonymous online postclass survey (Table 3). The 30 items were adapted from validated instruments: the Student Satisfaction and Self-Confidence in Learning scale and the Student Engagement scale [59,60], covering satisfaction, self-confidence, and engagement in learning. We also collected instructors’ insights through a survey with 6 open-ended questions. Full surveys are available in Multimedia Appendix 2. Participants were able to review and modify their responses before submission. Descriptive statistics were used to summarize questionnaire responses. Open-ended responses were analyzed using the 6-step thematic analysis approach, developed by Braun and Clarke [61]. One researcher read all responses repeatedly and generated initial codes. These codes were then grouped into preliminary themes. A second researcher subsequently reviewed the coding and the preliminary thematic structure. Any discrepancies in code interpretation or theme grouping were discussed between the 2 researchers until consensus was reached. Through this process, the final themes were named and synthesized.

**Table 3.** Structure of the postworkshop survey.

Participants, dimension, and subdimension			Number of items	Type
Students				
Satisfaction			5	5-point Likert scale
Engagement				
Behavioral engagement			5	5-point Likert scale
Emotional engagement			4	5-point Likert scale
Cognitive engagement			8	5-point Likert scale
Self-confidence			8	5-point Likert scale
Overall feedback			2	Open-ended questions
Instructors				
Overall feedback			6	Open-ended questions

### Ethical Considerations

The study was reviewed and approved by the institutional review board of Nanyang Technological University (IRB-2024‐928). Written informed consent was waived by the review board, as the study posed minimal risk and involved anonymous surveys conducted online. Online informed consent was provided to all participants prior to data collection, and participation was entirely voluntary. All survey data were collected anonymously with no personally identifiable information recorded, and access was restricted to the research team. Participants did not receive any compensation.

## Results

### Results of Study 1: Medical AI Competency Framework Development

#### Expert Validation and Framework Refinement

Most experts highlighted the importance of “AI foundations” (n=19) and “application skills” (n=23). These findings highlight the need for core AI knowledge and the ability to learn, apply, and co-develop AI solutions within medical workflows. As a medical professional emphasized, “gaining basic AI knowledge, gaining data analysis skills, and applying AI in clinical settings to improve patient care” were seen as essential competencies. Similarly, a hospital administrator noted that expected competencies include being “able to identify problems that can be solved by AI, understanding the process of deployment and evaluation of AI projects, applying AI knowledge in innovation, and possessing data analysis skills.”

In addition to technical competencies, 16 experts highlighted the importance of “critical reflections on AI,” emphasizing the ethical and safe use of AI in medicine. As a medical professional pointed out, “AI ethics concepts are similar to medical ethics concepts ... [professionals should] learn more about AI ethics and governance.” Moreover, 5 experts emphasized the integration of GenAI into clinical workflows. For example, a medical professional stated, “Using ChatGPT to conceptualize brief background of certain issues.” Based on expert feedback for greater clarity, “application skills” and “co-design equitable AI systems” were redefined to emphasize collaboration with engineers. For example, 1 hospital administrator highlighted:

*Not to become a coder/developer/engineer, but to get skillset to be able to comprehensively lead teams and run AI projects. Some technical + system thinking + appreciation of broader landscape.*

Furthermore, the definition of “real-world compliance” was expanded to stress the proper handling of both sensitive and nonsensitive data in clinical contexts. Detailed results are provided in Table S8 in Multimedia Appendix 3.

#### Final Medical AI Competency Framework

Table 4 and Table 5 show the final framework, which comprises 6 dimensions across 4 levels, ranging from foundational knowledge (know) to clinical practice (do).

**Table 4.** Medical artificial intelligence (AI) competency framework (part 1; dimensions 1‐3).

Aspects	Progression			
	Know	Know how	Show how	Do
Patient-centered AI^a^ in health care	Basic understanding: understand the foundational impact of AI on patient rights and autonomy Observable behaviors: identification of patient rights affected by AI use; recognition of patient autonomy concerns in AI-supported care; identification of patient-centered risks in AI use	Human advancement: learn how to apply AI to enhance treatment planning, automate care processes, and personalize health care for individual patients Observable behaviors: explanation of how AI supports treatment and personalization; identification of appropriate AI use in care processes	Patient-centered decision-making: use AI effectively to support decision-making, ensuring patient well-being is prioritized Observable behaviors: interpretation of AI outputs; comparison of AI recommendations with patient needs; justification of AI decisions based on patient well-being	Patient-centered practice: integrate AI tools into clinical practice, taking responsibility for incorporating them into patient care workflows to improve care quality and outcomes Observable behaviors: communication with patients about AI outputs in routine care workflows; integration of AI into patient care workflows with clinician oversight; accountable use of AI to support care quality
Ethics and transparency in clinical AI	Critical reflections on AI: comprehend the basic ethical principles guiding the use of AI in health care settings Observable behaviors: identification of core ethical principles in clinical AI; recognition of the importance of transparency in health care AI; recognition of ethical concerns in AI-supported care	Safe and responsible use: know how to use AI in ways that ensure ethical considerations and transparency Observable behaviors: explanation of ethical principles in health care AI use; consideration of transparency when planning AI-supported decisions; identification of ethical risks in planned AI use	Ethical decision-making: demonstrate ethical considerations during AI-enabled decision-making in simulated settings Observable behaviors: appraisal of ethical issues in simulated AI-supported cases; justification of decisions based on ethical and transparency considerations	Ethics by design: implement ethical standards in real-world AI applications, evaluating and improving systems for ethical compliance Observable behaviors: adherence to ethical standards in clinical AI practice; monitoring of AI use for ethical compliance; improvement of AI systems for ethical and transparent practice; documentation of ethical concerns in practice
Data privacy, security, and compliance in health care AI	Privacy awareness: remember regulations and the importance of safeguarding patient data Observable behaviors: awareness of privacy and data governance regulations; recognition of the importance of patient data protection	Regulatory adherence: know how to handle patient data securely, using techniques like anonymization and governance Observable behaviors: explanation of secure data handling practices; identification of data anonymization approaches	Secure data practices: manage patient data in simulated research projects, adhering to privacy laws Observable behaviors: application of data protection measures in structured tasks; management of data under privacy constraints in projects	Real-world compliance: process clinical data under specific conditions, ensuring all data handling and AI applications comply with privacy and security standards in clinical and research settings Observable behaviors: secure handling of clinical data in routine workflows; responsible data use in clinical and research settings

a AI: artificial intelligence.

**Table 5.** Medical artificial intelligence (AI) competency framework (part 2; dimensions 4-6).

Aspects	Progression			
	Know	Know how	Show how	Do
Technical proficiency in medical AI^a^ applications	AI foundations: acquire foundational knowledge of AI concepts and techniques Observable behaviors: awareness of core AI concepts; recognition of basic AI methods and models; understanding of algorithms in health care AI	Health care AI implementation: learn how to apply AI methods effectively in medical contexts Observable behaviors: explanation of AI applications in medical contexts; explanation of AI support for clinical decision-making; identification of appropriate clinical use cases for AI	Application skills: grasp the ability to perform and cocreate basic AI-related programming or data analysis tasks Observable behaviors: performance of basic AI-related programming tasks; performance of basic data analysis tasks	Evaluate and create AI: critically assess existing AI systems and develop solutions tailored to specific health care needs Observable behaviors: evaluation of AI systems for clinical relevance; development of AI solutions for health care needs; implementation of AI tools in real-world settings
Bias mitigation and health equity in AI design	Foundational understanding of bias: Identify how AI impacts health equity and its potential to either mitigate or exacerbate disparities Observable behaviors: awareness of bias in health care AI; recognition of AI-related health disparities; understanding of AI’s impact on health equity	Bias detection and analysis: know how to identify, evaluate, and address bias in AI systems within health care contexts Observable behaviors: explanation of bias sources in health care AI; evaluation of bias risks in AI use; identification of bias risks in data and AI systems	Equity-oriented practices: select and apply AI tools that minimize bias and enhance fairness, ensuring health equity in controlled design or assessment tasks Observable behaviors: selection of AI tools with fairness considerations; application of bias mitigation approaches in structured tasks	Co-design equitable AI systems: cocreate and refine AI solutions to enhance health equity, using iterative feedback to address systemic disparities Observable behaviors: co-design of AI solutions for health equity; refinement of AI systems to reduce bias; iterative improvement of AI for equitable practice
GenAI^b^ for health care	GenAI basics: identify the basic principles, limitations, and benefits of GenAI Observable behaviors: identification of core GenAI concepts; understanding of potential clinical uses of GenAI; recognition of GenAI limitations in health care	Interpretative proficiency: know how to interpret AI outputs, adapt data, and identify risks like hallucinations. Observable behaviors: Explanation of strategies for using GenAI tools; identification of hallucination and reliability risks	Skillful development: use GenAI, validate outputs, and communicate effectively in clinical scenarios Observable behaviors: validation of GenAI outputs in structured clinical tasks; writing of safe prompts; documentation of output limitations and need for human review	Integration in practice: independently integrates, evaluates, and oversees GenAI in real clinical workflows Observable behaviors: Integration of GenAI into real clinical workflows; verification of GenAI outputs before use in patient care; disclosure of limitations and maintenance of clinician oversight

a AI: artificial intelligence.

b GenAI: generative artificial intelligence.

### Translation of the Study 1 Findings Into the Study 2 Training Program

The final competency framework developed in study 1 served as the reference for the training program design in study 2. Specifically, 6 competency dimensions informed the learning topics and content of the program, while 4 Miller-aligned levels guided the design of learning objectives and assessment tasks. Learning activities were further developed in alignment with both the competency framework and educational theories. As shown in Table 6, the findings from study 1 were translated into the training program by mapping 6 dimensions of the medical AI competency framework into 5 modules. “Patient-centered AI in health care” and “ethics and transparency in clinical AI” were combined into a single module because of their close conceptual relationship in clinical practice. In this way, study 2 represents one practical application of the competency framework developed in study 1.

**Table 6.** Mapping of the medical artificial intelligence (AI) competency framework to the AI training program.

Module	Competency dimension	Learning focus
Module 1: patient-centered and ethical AI in Health Care	Patient-centered AI Ethics and transparency in clinical AI	Ethical principles of AI use in health care, patient autonomy, fairness, transparency, and responsible clinical decision-making
Module 2: AI privacy and security in health care	Data privacy, security, and compliance in health care AI	Data protection, regulatory frameworks (eg, HIPAA^a^ and GDPR^b^), and secure handling of health care data in AI applications
Module 3: medical AI applications	Technical proficiency in medical AI applications	Fundamental AI concepts, model implementation, and interpretation of AI outputs in health care contexts
Module 4: AI bias and health equity	Bias mitigation and health equity in AI design	Identification of bias in AI systems, fairness considerations, and strategies to promote equitable health care outcomes
Module 5: generative AI in Health Care	Generative AI for health care	Safe use of generative AI, including prompt design, output evaluation, and responsible integration into clinical workflows

a HIPAA: Health Insurance Portability and Accountability Act.

b GDPR: General Data Protection Regulation.

### Results of Study 2: Framework-Based Training Program Design and Feasibility Evaluation

#### Delphi Round 1 and Round 2 Results

Nine experts participated in both Delphi rounds. After round 1, consensus was reached on 16 of the 25 (64%) training program elements. The remaining 9 elements were carried forward to round 2 because they did not meet at least one of the predefined round 1 consensus criteria. In round 2, agreement was reached on the remaining 9 elements. Table 7 shows the results of the 2-round survey. Table S9 in Multimedia Appendix 3 shows the full results of the 2-round survey.

**Table 7.** Results of the 2-round Delphi study.

Element of program and module		Round 1^a^			Round 2^b^
		Median (IQR)	AS^c^ (%)	FS^d^ (%)	AS (%)
Teaching activities					
Module 3		4 (1)	88.89	44.44	100
Learning resources					
Module 1		5 (2)	66.67	66.67	100
Module 2		5 (2)	66.67	66.67	88.89
Module 5		5 (2)	66.67	55.56	88.89
Learning assessments					
Module 1		4 (1)	88.89	44.44	100
Module 2		4 (1)	88.89	44.44	100
Module 3		4 (1)	88.89	33.33	100
Module 4		4 (1)	88.89	44.44	100
Module 5		4 (1)	88.89	44.44	100

a In round 1, consensus was defined as meeting all the 3 criteria: IQR≤1, AS >75%, and FS >49.23%.

b In round 2, consensus was defined as achieving AS>80%.

c AS: agreement score proportion, the proportion of responses within a defined score range (eg, 4 and 5 on a 5-point scale) [53].

d FS: full score frequency, the proportion of the highest scores [54].

#### Final Framework–Based AI Training Program

**Figure 2.**
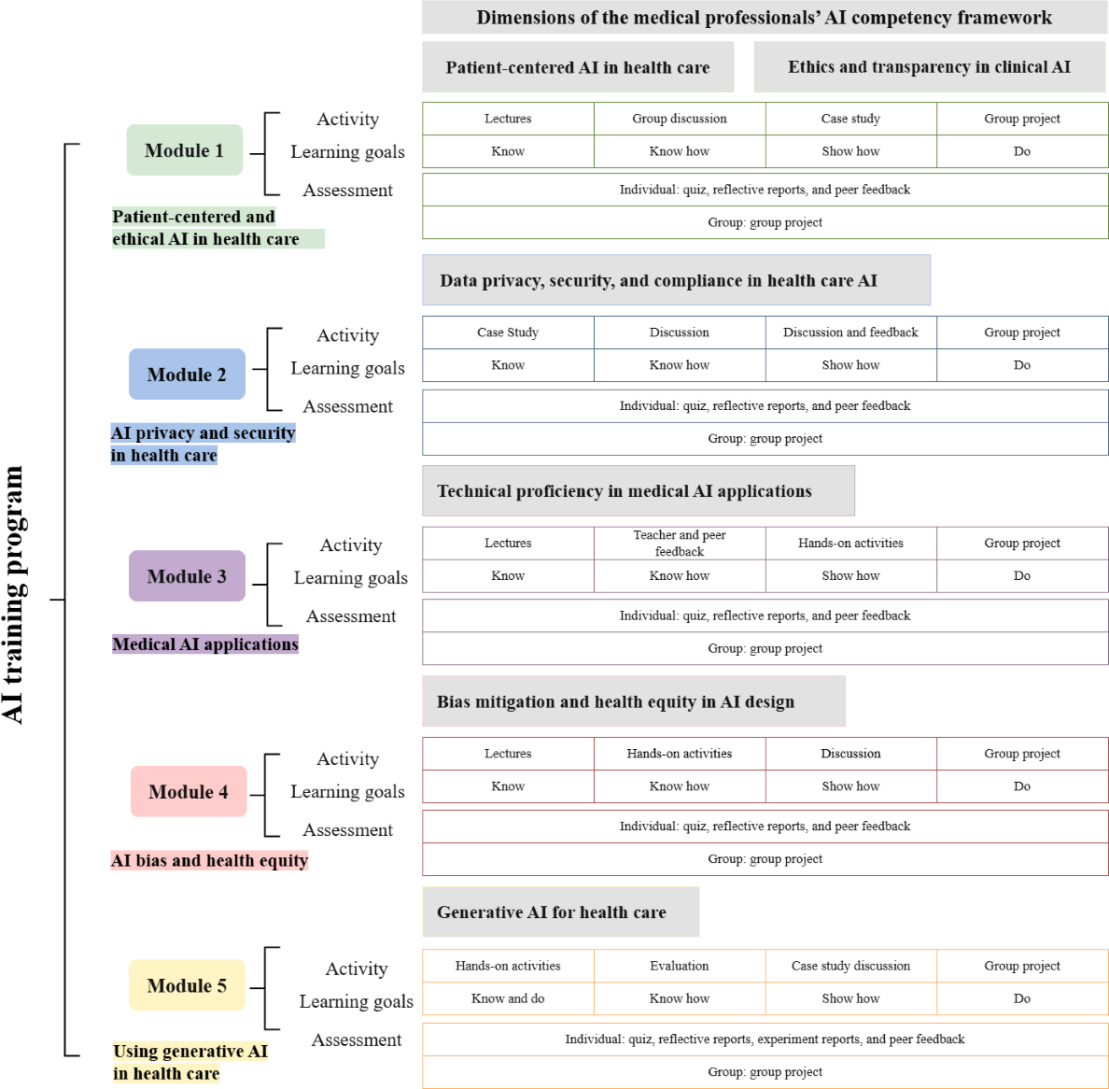
Structure of the artificial intelligence (AI) training program.

Figure 2 shows the overview structure of the 5-module AI training program. Multimedia Appendix 4 shows the final program design.

##### Module 1: Patient-Centered and Ethical AI in Health Care

This module aligns with the framework domains of “patient-centered AI in health care” and “ethics and transparency in clinical AI.” It introduces the ethical use of AI in health care, with a focus on patient-centered care, the impact of AI on patient rights, and the principles of transparency in clinical AI applications. Medical professionals are guided to balance ethical decision-making with AI integration, ensuring patient autonomy and the protection of patient rights. For example, Table 8 shows example learning tasks and assessments aligned with the “show how” and “do” levels of the Miller pyramid.

**Table 8.** Example alignment of learning tasks and assessment methods.

Module and level	Tasks	Assessment methods
Module 1		
Show how	Analyze a health care case involving AI^a^-supported diagnosis; identify issues related to patient autonomy, fairness, transparency, and human oversight; justify how patient interests should be protected.	Case analysis report or presentation with rubric-based assessment of ethical analysis, patient-centered health care, and justification of recommendations.
Do	Propose a proposal for an AI tool in health care that respects patient rights, supports fair decision-making, and includes clear human oversight in clinical use.	Group project with rubric-based assessment of patient-centered design, ethical safeguards, transparency, and practical integration into health care workflow.
Module 2		
Show how	Analyze privacy and security case studies involving improper disclosure, disposal, or handling of patient data.	Case-based task with structured rubric assessing identification of privacy and security risks.
Do	Design a health care AI project involving patient data; classify sensitive and nonsensitive data and propose protection measures.	Group project with rubric-based assessment of appropriateness of privacy and security measures and alignment with health care data governance requirements.
Module 3		
Show how	Run a preconfigured AI model using health care data; interpret performance metrics such as accuracy, *F*_1_-scores, precision, and recall; modify code or model settings (eg, hyperparameters).	Hands-on coding task with rubric-based assessment of code execution, interpretation of model outputs, and appropriateness of modifications.
Do	Adapt a basic AI model to a health care problem and present how the model could support clinical tasks, including its strengths and limitations.	Group project with rubric-based assessment of technical implementation, performance improvement, relevance to the health care problem, and critical interpretation of model limitations.
Module 4		
Show how	Use crafted imbalanced datasets to identify bias in model development; examine biased predictions using fairness metrics such as false positive rates.	Written reports assessed with structured rubric (bias detection, fairness analysis, and model interpretation).
Do	Design an AI model using a crafted dataset, apply bias mitigation methods such as oversampling, and evaluate whether the revised model improves fairness and equity.	Group project with rubric-based assessment of model design, bias mitigation, and fairness outcomes.
Module 5		
Show how	Write safe and effective prompts for clinical tasks; identify hallucinated or unsupported claims; analyze cases in which GenAI^b^ produces incorrect medical advice; document limitations explicitly.	Case-based task with a structured rubric assessing prompt quality, error detection, justification of validation steps, and documentation of limitations; individual report evaluating AI-generated outputs.
Do	Design a GenAI-supported solution based on a health care task (eg, documentation or literature synthesis); implement validation steps; specify when human review is required.	Group project with rubric-based assessment of use-case appropriateness, output reliability, validation strategy, transparency of limitations, and workflow integration.

a AI: artificial intelligence.

b GenAI: generative artificial intelligence.

##### Module 2: AI Privacy and Security in Health Care

This module corresponds to the framework domain of “data privacy, security, and compliance in health care AI.” It covers knowledge of data privacy, security, and regulatory compliance in medical AI applications. Medical professionals will learn key regulations (eg, General Data Protection Regulation and Health Insurance Portability and Accountability Act [HIPAA]) and ensure compliance in clinical settings. For example, Table 8 presents example learning tasks and assessments aligned with the “show how” and “do” levels of the Miller pyramid.

##### Module 3: Medical AI Applications

Aligned with the framework domain of “technical proficiency in medical AI applications,” this module provides knowledge of AI applications in health care. Medical professionals will learn the foundational principles of medical AI, explore AI applications, and gain hands-on experience in building and evaluating AI models. The module emphasizes application skills as well as the ability to evaluate and create clinically meaningful AI solutions. For example, Table 8 shows example learning activities and assessments aligned with the “show how” and “do” levels of the Miller pyramid.

##### Module 4: AI Bias and Health Equity

This module corresponds to the framework domain of “bias mitigation and health equity in AI design.” It develops medical professionals’ foundational understanding of algorithmic bias and its unequal impact on diverse patient populations. Through hands-on work with real datasets and explainable AI tools, they will examine how bias arises in data and affects models. The module further emphasizes bias detection, equity-oriented practices, and the co-design of fair and inclusive AI systems. For example, Table 8 shows example learning activities and assessments aligned with the “show how” and “do” levels of the Miller pyramid.

##### Module 5: Using Generative AI in Health Care

Aligned with the framework domain of “generative AI for health care,” this module introduces medical professionals to the principles and clinical applications of GenAI. They will acquire knowledge of generative models, learn prompt engineering, and gain practical experience applying GenAI. The module emphasizes the safe, effective, and responsible integration of GenAI into clinical practice. This emphasis is particularly important in health-related large language model applications, where outputs may be unverifiable or clinically incorrect, highlighting the need for validation, source grounding, and clinician oversight [21,62]. For example, Table 8 presents example learning activities and assessments aligned with the “show how” and “do” levels of the Miller pyramid.

### Pilot Implementation (Mini Workshop) Results

#### Overview

**Figure 3.**
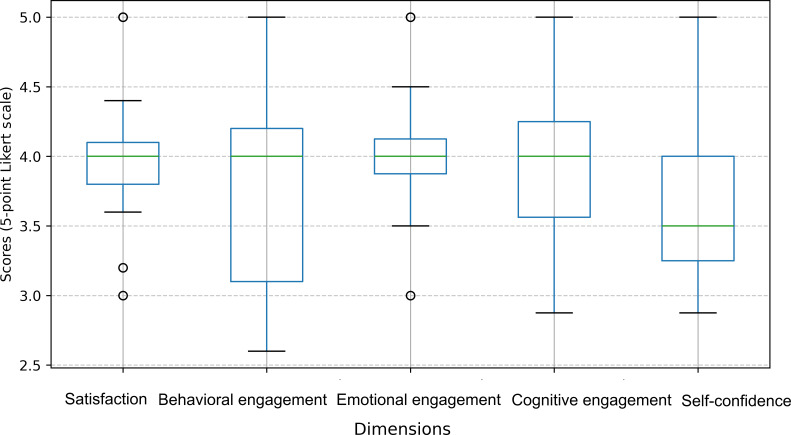
Distribution of 15 participant responses across the post–workshop survey dimension.

Of the 28 participants, 15 reported their learning experiences (Table 9). All questionnaires had high internal consistency, with Cronbach α values greater than 0.8. The mean scores were 4 (SD 0.52) for satisfaction, 3.8 (SD 0.71) for behavioral engagement, 4.05 (SD 0.51) for emotional engagement, 3.96 (SD 0.57) for cognitive engagement, and 3.63 (SD 0.53) for self-confidence (Table 10). Figure 3 illustrates the distribution of participant responses across survey dimensions.

**Table 9.** Participant descriptions and demographics.

Characteristics and categories	Participants (n=28), n (%)	Survey respondents (n=15), n (%)
Roles		
Year 2 medical student	12 (42.9)	6 (40)
Year 3 medical student	4 (14.3)	2 (13.3)
Year 4 medical student	7 (25)	4 (26.7)
Year 5 medical student	5 (17.9)	3 (20)
Training stage		
Preclinical (year 1-2)	12 (42.9)	6 (40)
Clinical (year 3-5)	16 (57.1)	9 (60)
Motivation for attending		
Voluntary	28 (100)	15 (100)
Others	0 (0)	0 (0)

**Table 10.** Descriptive statistics of post–workshop survey dimension (n=15).

Dimension	Mean (SD)	Median (IQR)	Min-max
Satisfaction	4 (0.52)	4 (5-3)	3.00-5.00
Behavioral engagement	3.80 (0.71)	4 (5-2.60)	2.60-5.00
Emotional engagement	4.05 (0.51)	4 (5-3)	3.00-5.00
Cognitive engagement	3.96 (0.57)	4 (5-2.88)	2.88-5.00
Self-confidence	3.63 (0.53)	3.50 (5-2.88)	2.88-5.00

Qualitative findings from open-ended responses revealed 3 main themes that reflected participants’ learning experiences and perceptions of the workshop.

#### Perceived Understanding of Coding and AI-Related Concepts

Participants reported learning coding basics, AI applications in medicine, and fundamental machine learning concepts. For instance, a participant described acquiring “a basic overview on AI, prompt generation, and machine learning.” Several participants also emphasized exposure to specific machine learning techniques, such as *“*oversampling, undersampling, and Shapley Additive Explanations plots.”

#### Development of Ethical and Reflective Awareness in AI Use

Beyond technical skills, participants demonstrated an increased awareness of ethical considerations and the reflective use of AI tools. Some participants mentioned learning about *“*AI ethics in medicine.” In addition, they reflected on how AI could support ongoing learning, with a participant noting learning *“*how to use AI tools to help with producing workable code and what concepts I need to be reading about for future self-learning.”

#### Learning Experience and Suggestions

Overall, students expressed positive perceptions of the learning environment, particularly valuing the instructors’ support and interactive teaching approach. Participants also offered constructive suggestions for improvement, such as covering “practical aspects of AI that can be applied to research projects.*”*

All instructors indicated that the “AI in Medicine” training workshop was well received, engaged students, and largely achieved its learning objectives, although some complex content required additional time. While participants enjoyed the workshop, their confidence in applying AI remained limited. For example, an instructor noted:

*Participants demonstrated strong engagement and interest throughout the workshops. They consistently asked questions, ... , they found some of the machine learning content challenging. They asked numerous questions related to the depth of the ML material, which suggests that time constraints limited their ability to fully absorb these concepts. Several participants expressed a desire for additional time to gain a deeper understanding of the machine learning and explainable AI methods covered in the sessions.*

Instructors also offered several suggestions for future implementations, including providing hands-on and practical sessions (emphasizing experiential learning and group activities, as these are the most engaging and effective), simplifying and allocating more time (simplifying technical content for nontechnical participants and providing more time and clearer instructions for practical exercises), and changing content depth and focus (narrowing the range of machine learning methods and exploring key topics in greater depth).

These self-reported findings align with several topics identified in study 1’s framework, particularly the basic understanding of AI concepts and their application in health care.

## Discussion

### Principal Results

This study addresses a critical gap in global medical education: the absence of standardized medical AI curriculum frameworks [30,31]. By integrating the UNESCO AI competency framework with the Miller pyramid, we translated high-level AI literacy principles into clinically actionable competencies across 6 dimensions. The 6D framework provides a structured reference for competency development, spanning foundational AI understanding to ethical, patient-centered, and practice-integrated application.

Building on this framework, we translated these competencies into a structured training program and piloted a workshop. The pilot implementation of module 3 provided initial evidence of the program’s feasibility, although the self-reported learning experience and confidence required further evaluation.

### Comparison With Prior Work

Extending UNESCO’s AI competency framework to the medical domain [17], we incorporated domain-specific needs, such as patient-centered decision-making, ethical considerations in clinical AI, and health equity. Consistent with prior studies, our findings reinforce the importance of integrating both technical (eg, AI applications) and nontechnical competencies (eg, ethics) [11,12,15,35,36]. Importantly, this study responds to the growing impact of GenAI in health care by integrating competencies for its responsible use. Although recent literature has discussed both the applications and risks of GenAI in clinical contexts [21-23], few studies have articulated the competencies required for medical professionals to use these technologies safely and effectively. The inclusion of GenAI competencies provides a forward-looking perspective that is currently insufficiently examined in existing research.

Prior studies on AI education in medicine have been largely domain-specific, focusing on areas such as machine learning fundamentals or programming [26,27,29,42]. For example, some programs emphasized conceptual understanding of AI methods through lectures [26,29], while others focused on developing basic programming skills [25]. Although these initiatives contribute to increased awareness and technical competence, they are typically organized around discrete topics rather than being guided by a unified framework. Furthermore, the literature has highlighted the absence of unified competency frameworks to guide these efforts [30,31]. In contrast, our study presents a framework-guided curriculum design approach that translates competency domains into training programs.

### Theoretical and Educational Implications

**Figure 4.**
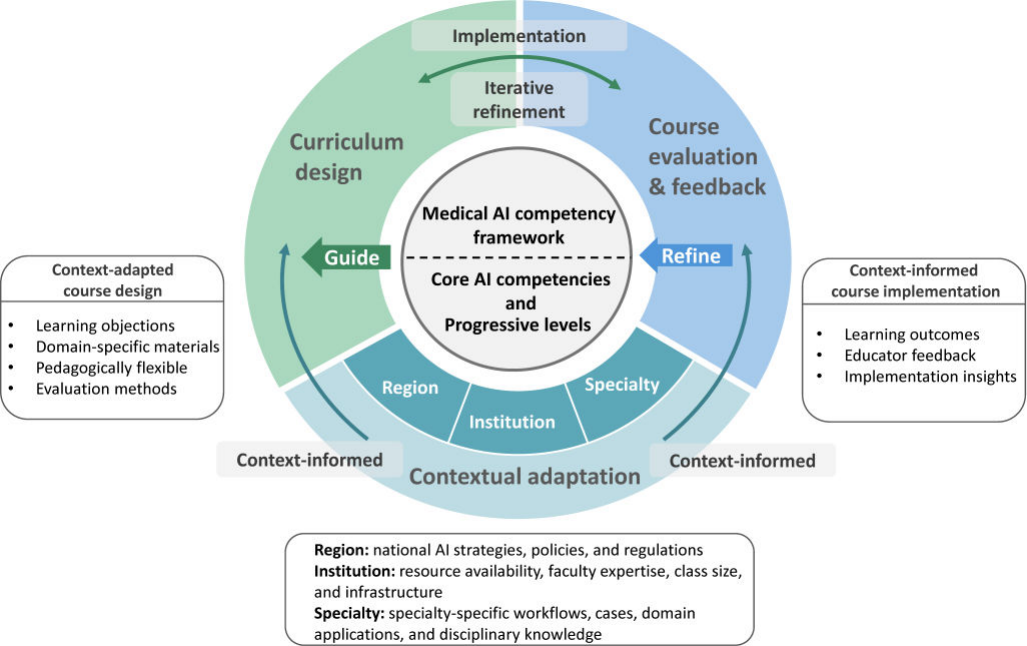
The medical artificial intelligence (AI) competency framework as a living framework.

This study provides both theoretical and practical implications. Theoretically, it proposes a medical AI competency framework that defines shared foundational AI competencies for medical education. It focuses on how medical professionals interact with, evaluate, communicate, and apply AI in health care, rather than replacing discipline-specific knowledge. We, therefore, view it as a living framework (Figure 4). While it defines core competencies, its implementation should be tailored to regional, institutional, and specialty-specific contexts. Such adaptation occurs mainly at the curriculum level. For example, different specialties may require different cases, data, and learning materials. Feedback from course implementation can then support the iterative refinement of both the curriculum and the framework.

Practically, the framework serves as a reference for curriculum design. By linking competency domains with the Miller levels, this framework supports curriculum mapping, the design of learning objectives and assessments, and the identification of training gaps. Additionally, prior work suggests that AI governance cannot rely solely on external constraints [9]. In this context, our framework supports the development of competencies for responsible AI use and may contribute to a broader governance-oriented approach to AI adoption in health care. This study further presents a competency-based AI training program that may serve as a model for other institutions. In addition, the explicit integration of GenAI into the competency framework and curriculum is particularly timely, given its rapid adoption in health care, education, and research.

### Limitations and Future Directions

There are several limitations to this study. First, although the experts in this study represented diverse backgrounds, the majority were based in Singapore. As cultural attitudes toward AI adoption differ significantly across regions [63], the framework may mainly reflect Singapore’s specific health care and educational context. The framework should be viewed as a living framework, open to ongoing refinement. Future research can validate and extend it using more diverse expert samples and combined deductive-inductive approaches. Future work may also extend the framework to specialty-specific applications (eg, course design and implementation) in areas such as radiology, surgery, and public health, using locally relevant cases, datasets, and assessment methods.

Second, the pilot was conducted as a voluntary workshop in Singapore, which may limit generalizability. Participants may have had greater prior interest in AI, introducing self-selection bias and potentially inflating the reported results. In addition, the pilot evaluation was based only on self-reported measures, covered only 1 module, and had a modest response rate (15/28), which may introduce nonresponse bias. Accordingly, these findings should be interpreted as preliminary evidence of feasibility. Future studies should evaluate the full program in broader settings using preassessments and postassessments of competency gains.

Third, while our program emphasizes real-world applicability, its short-term nature limits the direct assessment of AI competencies in clinical practice. Future work should collaborate with health care institutions to integrate training into clinical workflows and examine the longer-term impact across multiple levels, for example, using the Kirkpatrick model [64].

### Conclusions

We developed a theoretically grounded medical AI competency framework. By translating competencies into a structured training program, it provides a practical application for integrating AI into medical education. Ultimately, this work aims to contribute to the development of an AI-ready health care workforce capable of using AI safely, ethically, and effectively in clinical practice.

## Supplementary material

10.2196/91116 Multimedia Appendix 1 Initial medical artificial intelligence (AI) competency framework and codebook for deductive content analysis.

10.2196/91116 Multimedia Appendix 2 Course evaluation surveys and post–workshop surveys.

10.2196/91116 Multimedia Appendix 3 Deductive coding and Delphi results.

10.2196/91116 Multimedia Appendix 4 Final artificial intelligence (AI) training program.

## Abbreviations

AI: artificial intelligence
AS: agreement score
FS: full score
GenAI: generative artificial intelligence
HIPAA: Health Insurance Portability and Accountability Act
UNESCO: United Nations Educational, Scientific and Cultural Organization
