# Supplementary material for: A Competency Framework for Medical AI Education: Mixed Methods Study
Source: JMIR Med Educ. 2026 May 20;12:e91116. doi: 10.2196/91116 (PMC13189368; doi:10.2196/91116)
Supplement: Multimedia Appendix 1 [file mededu-v12-e91116-s001.docx]

**Multimedia Appendix 1**

**Table S1. Initial medical AI competency framework.**

| **Aspects** | **Progression** | | | |
| --- | --- | --- | --- | --- |
|  | **Know** | **Know how** | **Show how** | **Do** |
| **Patient-centered AI in health care** | **Basic understanding:**  Understand the foundational impact of AI on patient rights and autonomy. | **Human advancement:** Know how to use AI for better care planning. | **Patient-centered decision-making:** Use AI effectively to support decision-making, ensuring patient well-being is prioritized. | **Patient-centered practice:** Integrate AI tools in clinical practice, ensuring patient-centric, ethical care while guiding treatment decisions. |
| **Ethics and transparency in clinical AI** | **Critical reflections on AI:** Comprehend the basic ethical principles guiding the use of AI in health care settings. | **Safe and responsible use:** Know how to use AI in ways that ensure ethical considerations and transparency. | **Ethical decision-making:** Demonstrate ethical considerations during AI-enabled decision-making in simulated settings. | **Ethics by design:** Implement ethical standards in real-world AI applications, evaluating and improving systems for ethical compliance. |
| **Data privacy, security, and compliance in health care AI** | **Privacy awareness:** Remember regulations and the importance of safeguarding patient data. | **Regulatory adherence:** Know how to handle patient data securely, employing techniques like anonymization and governance. | **Secure data practices:** Manage patient data in simulated research projects, adhering to privacy laws. | **Real-world compliance:** Ensure all data processing and AI applications meet privacy and security requirements in clinical and research environments. |
| **Technical proficiency in medical AI applications** | **AI foundations:** Acquire foundational knowledge of AI concepts and techniques. | **Health care AI implementation:** Learn how to apply AI methods effectively in medical contexts. | **Application skills:** Grasp the ability to perform basic AI-related programming or data analysis tasks. | **Evaluate and create AI:** Develop and implement advanced AI solutions for solving complex clinical challenges, showcasing technical expertise in real scenarios. |
| **Bias mitigation and health equity in AI design** | **Foundational understanding of bias:** Identify how AI impacts health equity and its potential to either mitigate or exacerbate disparities. | **Bias detection and analysis:** Know how to identify, evaluate, and address bias in AI systems within health care contexts. | **Equity-oriented practices:** Select and apply AI tools that minimize bias and enhance fairness, ensuring health equity in controlled design or assessment tasks. | **Design equitable AI systems:** Refine AI solutions to enhance health equity, using iterative feedback to address systemic disparities. |
| **Generative AI for health care** | **Generative AI basics:** Identify the basic principles, limitations, and benefits of GenAI. | **Interpretative proficiency:** Know how to interpret AI outputs, adapt data, and identify risks like hallucinations. | **Skillful development:** Use or design generative AI tools in structured or project-based settings. | **Integration in practice:** Incorporate generative AI into clinical workflows. |

**Codebook for Deductive Content Analysis**

Tables S2–S7 present the codebooks used for deductive content analysis. Each codebook corresponds to one competency dimension and the four proficiency levels of Miller’s pyramid.

Table S2. Codebook: patient-centered AI in health care

| Level | Definition | Indicators |
| --- | --- | --- |
| Know | Applied when experts referred to basic awareness or understanding of the foundational impact of AI on patient rights and autonomy. | General concerns about the use of AI in medicine or patient care; awareness of potential impacts on patient rights and autonomy. |
| Know how | Applied when experts described how AI can be used to enhance treatment planning, automate care processes, and personalize health care for individual patients. | Using AI to support treatment planning, automate aspects of care, or tailor health care to individual patient needs. |
| Show how | Applied when experts described how to use AI effectively to support decision-making, ensuring patient well-being is prioritized. | Application of AI for clinical decision tasks, such as risk stratification or prediction of patient condition changes to inform care planning. |
| Do | Applied when experts referred to the use of AI in real-world clinical practice, taking responsibility for incorporating them into patient care workflows to improve care quality and outcomes. | Integration of AI tools into clinical workflows; accountable use of AI in everyday clinical practice to improve patient care and outcomes. |

Table S3. Codebook: ethics and transparency in clinical AI

| Level | Definition | Indicators |
| --- | --- | --- |
| Know | Applied when experts referred to foundational knowledge or awareness of basic ethical principles guiding the use of AI in health care settings. | Awareness or understanding of AI ethics; references to ethical concerns or the importance of ethics in health care AI. |
| Know how | Applied when experts described recognizing how ethical principles and guidelines should be applied to AI systems used in health care contexts. | Application of ethical guidelines or frameworks to health care AI scenarios; consideration of ethics when planning or evaluating AI use. |
| Show how | Applied when experts referred to ethical considerations during AI-enabled decision-making in simulated settings. | Critical appraisal or analysis of AI proposals, projects, or outputs with attention to ethical implications. |
| Do | Applied when experts referred to applying ethical standards in real-world AI practice, including evaluating and improving systems for ethical compliance. | Adherence to ethical standards in clinical practice; monitoring, evaluating, or improving AI systems to ensure ethical compliance in practice. |

Table S4. Codebook: data privacy, security, and compliance in health care AI

| Level | Definition | Indicators |
| --- | --- | --- |
| Know | Applied when experts referred to basic understanding of data protection regulations and the importance of safeguarding patient data in health care AI. | Awareness of data governance and privacy regulations (e.g., HIPAA, GDPR); recognition of the importance of patient data protection. |
| Know how | Applied when experts described how patient data should be handled securely and how data quality should be evaluated in health care AI contexts. | Secure data handling practices; data anonymization; evaluation of data quality or suitability for AI use. |
| Show how | Applied when experts referred to managing patient data in structured or simulated projects in accordance with privacy laws and ethical standards. | Managing datasets under privacy constraints; applying data protection measures in simulated research or development settings. |
| Do | Applied when experts referred to ensuring privacy, security, and compliance in real-world clinical settings. | Routine compliance with data privacy and security requirements; data handling in real-world workflows. |

Table S5. Codebook: technical proficiency in medical AI applications

| Level | Definition | Indicators |
| --- | --- | --- |
| Know | Applied when experts referred to foundational knowledge of core AI concepts and techniques relevant to health care. | Awareness of basic AI concepts or terminology; references to foundational AI knowledge such as algorithms, data, or models. |
| Know how | Applied when experts described how AI methods can be applied effectively in medical or health care contexts. | Mentions of using AI for data analysis, clinical decision support, or diagnostic assistance. |
| Show how | Applied when experts referred to demonstrating technical skills related to AI, such as programming, data analysis, or model use. | Performing basic AI-related programming or data analysis tasks. |
| Do | Applied when experts referred to developing, implementing, or routinely using AI solutions to address real-world clinical challenges. | Developing or deploying AI tools in clinical practice; designing AI systems to support treatment, diagnosis, or patient management in real-world settings. |

Table S6. Codebook: bias mitigation and health equity in AI design

| Level | Definition | Indicators | |
| --- | --- | --- | --- |
| Know | Applied when experts referred to awareness of how AI systems may influence health equity, including their potential to introduce or amplify bias. | | Awareness of AI bias; recognition of disparities or inequities arising from AI use; general references to fairness or health equity in AI. |
| Know how | Applied when experts described how bias in AI systems can be identified, evaluated, or addressed in health care contexts. | | Identifying sources of bias in data or models; assessing bias in AI outputs. |
| Show how | Applied when experts referred to the selection of AI tools and methods that minimize bias and promote fairness in structured or simulated tasks. | | Evaluating or selecting AI solutions; applying bias mitigation approaches. |
| Do | Applied when experts referred to improving or refining AI systems in real-world practice to enhance health equity and address systemic disparities. | | Routine efforts to reduce bias in deployed AI systems; iterative refinement of AI solutions to promote equity in clinical practice. |

Table S7 Codebook: generative AI for health care

| Level | Definition | Indicators |
| --- | --- | --- |
| Know | Applied when experts referred to awareness of the capabilities, limitations, and potential applications of generative AI in health care. | Awareness of generative AI concepts; references to basic functions, strengths, or limitations of large language models or other generative systems. |
| Know how | Applied when experts described how generative AI tools can be used effectively to support health care tasks. | Use of generative AI tools through strategies; mention of prompt engineering. |
| Show how | Applied when experts referred to demonstrating the use or design of generative AI tools in structured or project-based settings. | Applying generative AI or large language models to specific tasks; adapting generative AI tools for health care use case. |
| Do | Applied when experts referred to integrating generative AI into real-world health care workflows in a responsible manner. | Routine use of generative AI to support clinical tasks (e.g., documentation, summarization, transcription) within health care workflows. |
