# Supplementary material for: A Competency Framework for Medical AI Education: Mixed Methods Study
Source: JMIR Med Educ. 2026 May 20;12:e91116. doi: 10.2196/91116 (PMC13189368; doi:10.2196/91116)
Supplement: Multimedia Appendix 2 [file mededu-v12-e91116-s002.docx]

**Multimedia Appendix 2**

**Course Evaluation Surveys**

Round 1 survey, which contains the original course design, is available here:

https://drive.google.com/file/d/1-W-2x81m4QLaCLyCxQ4ipBgtpIwMfAeM/view?usp=sharing.

Round 2 survey can be accessed here: https://drive.google.com/file/d/1L6HxOLrloGZD2FJtSFOJxcesPDFk8ezb/view?usp=sharing.

**Post-Workshop Surveys**

The post-workshop survey used to collect student feedback can be accessed here: https://docs.google.com/document/d/18YdvKWXrinbZQjdT1qAyaDj5q6SiM2du/edit?usp=sharing&ouid=101986732616285029116&rtpof=true&sd=true.

The post-workshop survey used to collect instructors’ feedback can be accessed here: https://docs.google.com/document/d/1lAnWABaNvm3mxC8Gks9pJJJPUXrRwjWxQjPa9msNfRA/edit?usp=sharing.
