# Supplementary material for: A Competency Framework for Medical AI Education: Mixed Methods Study
Source: JMIR Med Educ. 2026 May 20;12:e91116. doi: 10.2196/91116 (PMC13189368; doi:10.2196/91116)
Supplement: Multimedia Appendix 3 [file mededu-v12-e91116-s003.docx]

**Multimedia Appendix 3**

**Deductive Coding Results**

Table S8. Expert Coding Results Supporting Framework Validation

| Aspects | | Progression | | | |  |
| --- | --- | --- | --- | --- | --- | --- |
|  |  | Know | Know how | Show how | Do | |
| Patient-Centered AI in Healthcare | 4 (16.67%) | | 5 (20.83%) | 5 (20.83%) | 7 (29.17%) | |
| Ethics and Transparency in Clinical AI | 16 (66.67%) | | 5 (20.83%) | 4 (16.67%) | 2 (8.33%) | |
| Data Privacy, Security, and Compliance in Healthcare AI | 7 (29.17%) | | 2 (8.33%) | 2 (8.33%) | 4 (16.67%) | |
| Technical Proficiency in Medical AI Applications | 19 (79.17%) | | 18 (75.00%) | 23 (95.83%) | 10 (41.67%) | |
| Bias Mitigation and Health Equity in AI Design | 3 (12.50%) | | 3 (12.50%) | 6 (25.00%) | 2 (8.33%) | |
| Generative AI for Healthcare | 5 (20.83%) | | 2 (8.33%) | 1 (4.17%) | 5 (20.83%) | |

Note. Values indicate the number and percentage of experts whose responses were coded under each competency dimension and progression level using deductive content analysis (n = 24). A single expert response could be coded into multiple categories.

**Two-Rounds Delphi Results**

Table S9. Full Results of the Two-Rounds Delphi Study

|  |  | Round 1 | | | Round2 | | |
| --- | --- | --- | --- | --- | --- | --- | --- |
| Element of Program | Module | IQR | AS (%) | FS (%) | IQR | AS (%) | FS(%) |
| Course Content | Module 1 | 1 | 100 | 66.67 | - | - | - |
|  | Module 2 | 0 | 88.89 | 77.78 | - | - | - |
|  | Module 3 | 0 | 100 | 77.78 | - | - | - |
|  | Module 4 | 0 | 100 | 88.89 | - | - | - |
|  | Module 5 | 0 | 88.89 | 77.78 | - | - | - |
| Learning Goals | Module 1 | 1 | 88.89 | 66.67 | - | - | - |
|  | Module 2 | 1 | 88.89 | 66.67 | - | - | - |
|  | Module 3 | 1 | 88.89 | 66.67 | - | - | - |
|  | Module 4 | 0 | 88.89 | 77.78 | - | - | - |
|  | Module 5 | 1 | 88.89 | 66.67 | - | - | - |
| Teaching Activities | Module 1 | 1 | 88.89 | 55.56 | - | - | - |
|  | Module 2 | 1 | 88.89 | 66.67 | - | - | - |
|  | Module 3 | 1 | 88.89 | 44.44 | 0 | 100 | 77.78 |
|  | Module 4 | 0 | 88.89 | 77.78 | - | - | - |
|  | Module 5 | 1 | 88.89 | 66.67 | - | - | - |
| Learning Resources | Module 1 | 2 | 66.67 | 66.67 | 0 | 100 | 77.78 |
|  | Module 2 | 2 | 66.67 | 66.67 | 1 | 88.89 | 55.56 |
|  | Module 3 | 1 | 88.89 | 66.67 | - | - | - |
|  | Module 4 | 1 | 88.89 | 66.67 | - | - | - |
|  | Module 5 | 2 | 66.67 | 55.56 | 1 | 88.89 | 44.44 |
| Learning Assessments | Module 1 | 1 | 88.89 | 44.44 | 0 | 100 | 77.78 |
|  | Module 2 | 1 | 88.89 | 44.44 | 0 | 100 | 77.78 |
|  | Module 3 | 1 | 88.89 | 33.33 | 0 | 100 | 77.78 |
|  | Module 4 | 1 | 88.89 | 44.44 | 0 | 100 | 77.78 |
|  | Module 5 | 1 | 88.89 | 44.44 | 0 | 100 | 77.78 |

Note: In Round 1, consensus was defined as meeting all three criteria: IQR ≤ 1, AS >75%, and FS > 49.23%. In Round 2, consensus was defined as achieving AS > 80%.
