# Supplementary material for: A Competency Framework for Medical AI Education: Mixed Methods Study
Source: JMIR Med Educ. 2026 May 20;12:e91116. doi: 10.2196/91116 (PMC13189368; doi:10.2196/91116)
Supplement: Multimedia Appendix 4 [file mededu-v12-e91116-s004.docx]

**Multimedia Appendix 4**

**Final AI Training Program**

The final course design is available here: https://drive.google.com/file/d/14rmaVyzrhbpG2xysWuu6q9Xzd1Hrgp16/view?usp=sharing.
